# ICESat-2 land ice products resolve Greenland and Antarctic ice-sheet height changes on seasonal to multiyear time scales

**DOI:** 10.1017/jog.2026.10152

**Published:** 2026-03-24

**Authors:** Benjamin E. Smith, Tyler Clark Sutterley, Helen A. Fricker, Laurie Padman, Matthew R. Siegfried, Taryn Black, Denis Felikson, Bryony Freer, Aimée Gibbons, Susan L. Howard, Benjamin Jelley, Michalea King, Brooke Medley, Mathieu Morlighem, Christine Sadlik, Wilson Sauthoff, Thomas A. Neumann

**Affiliations:** 1 Polar Science Center, Applied Physics Laboratory, University of Washington, Seattle, WA, USA; 2 Scripps Institution of Oceanography, University of California, San Diego, La Jolla, CA, USA; 3 Earth and Space Research, Corvallis, OR, USA; 4 Hydrologic Sciences & Engineering Program, Department of Geophysics, Colorado School of Mines, Golden, CO, USA; 5 Earth System Science Interdisciplinary Center, University of Maryland, College Park, MD, USA; 6 Cryospheric Sciences Laboratory, NASA Goddard Space Flight Center, Greenbelt, MD, USA; 7 KBR Inc., Greenbelt, MD, USA; 8 Earth and Space Research, Seattle, WA, USA; 9 Earth Sciences Division, NASA Goddard Space Flight Center, Greenbelt, MD, USA; 10 Department of Earth Sciences, Dartmouth College, Hanover, NH, USA

**Keywords:** glacier mapping, glacier monitoring, laser altimetry, remote sensing

## Abstract

NASA’s ICESat-2 mission was launched in 2018, carrying a photon-counting laser altimeter, with a primary objective of measuring height changes across Earth’s surface. ICESat-2 has provided measurements of ice surface height between 88º N and S, repeated four times per year, with high vertical accuracy and along-track spatial resolution. Its accuracy and coverage has enabled near-complete recovery of height changes across the ice sheets, capturing subtle changes in the interior, and rapid changes along the dynamic margins with steep slopes and the floating peripheral ice shelves. The ICESat-2 Science Team has developed a suite of algorithms that produce along-track and gridded land ice height products at various levels of processing, all freely available at the National Snow and Ice Data Center. Here, we describe three higher-level land-ice data products derived from ATL06 and their underlying algorithms: along-track height change (ATL11), digital elevation model (ATL14) and gridded surface height change (ATL15). We demonstrate the suitability of each data product for studying different ice sheet regions. We then show height changes for Greenland and Antarctica from ATL15 during the first 6 years of the ICESat-2 mission (October 2018–December 2024), illustrating how ICESat-2 measurements can distinguish the multi-year trends from seasonal fluctuations.

## 1. Introduction

Earth’s glaciers, ice caps and ice sheets (together termed ‘land ice’) are critical components of the climate system and water cycle, with changes in their mass directly contributing to global sea level change. Sea level rise averaged 3.61 mm a${}^{-1}$ between 2006 and 2018, with 17% from loss of land water storage, 38% from ocean thermal expansion and 45% from land ice melt (Fox-Kemper and others, 2021). The contributions from Greenland and Antarctica have both increased since the 1990s, by factors of 5 and 1.25, respectively (Otosaka and others, 2023), highlighting the importance of continued monitoring. Three satellite techniques track land ice mass changes: gravimetry (primarily from the Gravity Recovery and Climate Experiment, GRACE, and its Follow-On, GRACE-FO) to directly measure mass variations; synthetic aperture radar (SAR) combined with ice thickness and climate models to estimate mass flux; and altimetry (both radar and laser) integrated with firn density models to infer mass changes. The Ice Mass Balance Intercomparison Exercise, a joint European Space Agency (ESA) and NASA project, combines output from these techniques for robust assessments (Otosaka and others, 2023). Gravimetry directly measures mass change (e.g., Chen and others, 2006; Velicogna and Wahr, 2006), but has coarse spatial resolution (> 100 km) (Tapley and others, 2019). Mass flux estimates typically provide an assessment at the glacier catchment level (e.g., Joughin and Tulaczyk, 2002; Rignot and others, 2019), but rely on precise knowledge of the ice thickness at the flux gate, which is poorly sampled in some key regions (MacGregor and others, 2021). The third method, satellite altimetry, uses radar or laser altimeters to infer the mass change from the measured surface height change, factoring in ice and snow density variations (e.g., Wingham and others, 1998; Smith and others, 2020). Although altimetry-based methods require detailed knowledge of surface snow and firn density to estimate mass change, they can provide much higher spatial resolution of changes than the other methods, resolving key individual features like glaciers and ice streams (e.g., Medley and others, 2022; van den Broeke and others, 2023). The two types of altimetry sensors offer complementary views of mass change. Radar altimetry is unaffected by clouds, but the signal penetrates the surface snow layer to a poorly constrained depth, and radar footprints are wider than the length scales of some important land ice surface features (Fricker and others, 2010; Magruder and others, 2024). Laser altimetry faces data loss from clouds but measures heights very close to the true snow surface (Fair and others, 2024; Smith and others, 2025a) with a smaller footprint and at a high along-track resolution (Neumann and others, 2023; Smith and others, 2023e).

NASA has flown two polar-orbiting satellite laser altimeter missions. ICESat (Schutz and others, 2005) operated from 2003 to 2009, using the Geoscience Laser Altimeter System, a single-beam near-infrared (1064 nm) full-waveform laser altimeter in a near-polar orbit (86º S to 86º N). It collected data in campaign mode along 30 day subcycles of a 91 day orbit, 2–3 times per year. Because ICESat was a single-beam instrument and its measurements were spread as much as 30–40 m around the repeat tracks, there was ambiguity between the across-track surface slope and the elevation change, which in some ice-sheet regions limited accurate interpretation of the ICESat time series. ICESat-2 (Markus and others, 2017) was launched in September 2018, carrying the Advanced Topographic Laser Altimeter System (ATLAS), a green (532 nm) photon-counting laser altimeter with a six-beam design to increase spatial sampling, and cross-track slope detection. With a sampling rate of 0.7 m along-track and a footprint of ~11 m (Magruder and others, 2021), ICESat-2 can retrieve cm-scale height changes averaged on ~100 m along-track scales (Brunt and others, 2021). ICESat-2 ground tracks also repeat at ~91 day intervals, between 88º S and 88º N latitude. Its repeating tracks have smaller deviations than ICESat tracks because of improved pointing control and improved calibration of the spacecraft pointing system (Luthcke and others, 2021). A previous study (Smith and others, 2020) used crossover data between the ICESat and ICESat-2 missions to make the first comprehensive map of surface height change and estimated mass balance over the Antarctic and Greenland ice sheets, including floating ice shelves. These results confirmed ongoing mass loss from both ice sheets during 2003–19, updating earlier laser-altimetry based estimates (Pritchard and others, 2009, 2012), and revealed a widespread link between floating ice loss and reduced buttressing of grounded ice, previously seen only locally (Scambos and others, 2004) or in models (Gudmundsson and others, 2019). Smith and others (2020) also found that temporal variability reflects competing oceanic and atmospheric influences. However, the spatial sampling of height change in that study was limited by the use of spatially sparse inter-mission crossovers and by using only the first year of ICESat-2 data. After over 6 years in orbit, ICESat-2 data now allow identification of the multi-year trend within a single mission and resolution of seasonal and annual fluctuations (e.g., Adusumilli and others, 2022; Taubenberger and others, 2022).

ICESat-2 provides global surface height data to monitor essential climate variables, including ice sheet volume changes, sea ice thickness and biomass (Magruder and others, 2024). In this paper, we review the ICESat-2 products available through the National Snow and Ice Data Center (NSIDC) for land ice change detection at a range of spatial and temporal scales. The suite of data products from ATLAS on ICESat-2 is designated ATL*xx*, with *xx* being a two-digit product code. We first summarize ICESat-2’s orbit and sampling characteristics and review the Level-2 global geolocated photon data product (ATL03; Neumann and others, 2019) and Level-3A along-track land-ice height product (ATL06; Smith and others, 2019). We then describe the methods used to generate the Level-3B along-track slope-corrected land-ice height-change time series product (ATL11) and gridded land-ice height (ATL14) and height-change (ATL15) products. We explain the sequence by which these data products are processed from ATL03 data and demonstrate the uses of each product for examining changes in ice-sheet height. These examples are intended to provide users of ICESat-2 land ice data with guidance on product choice for their application. We then present ATL15 height-change maps that resolve dynamically and meteorologically driven processes at quarter-annual resolution. Trends and seasonal signals over multiple years at the basin scale will be invaluable input for initializing and validating ice sheet models for improving projections of sea level change.

## 2. Mission background and lower-level data products

### 2.1. ICESat-2 mission overview

**Figure 1.**
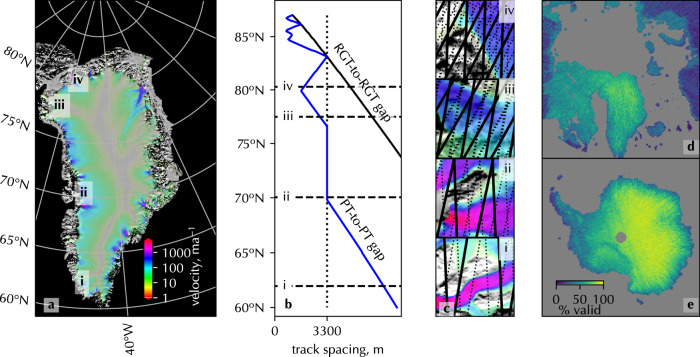
Location map (a) shows ice speed (colors, Joughin, 2023) superimposed over an image mosaic of Greenland (grayscale, Haran and others, 2018). ICESat-2 RPT sampling across Greenland (b) is expressed as the largest gap between adjacent RPTs for any latitude. The RGT-to-RGT spacing is shown for reference. 10 × 10 km maps for four select locations (c) show the pattern of tracks, with RGTs shown in solid lines and RPTs shown in dashed lines. Maps of the fraction of repeat tracks for which surface returns were observed, for the (d) Arctic and (e) Antarctic from April 2019 to September 2025. Repeat counts are derived from the number of valid measurements in the ATL11 product (see Section 3.1). Over Greenland, the Arctic ice caps and northeastern Canada, the repeat fraction is related to the loss of returns due to clouds. In other parts of the Arctic, the repeat fraction is driven largely by the number of measurements targeted at the repeat tracks. Figure 1 long description.

The ATLAS instrument on ICESat-2 has two lasers, of which one is operational and the other is reserved as a backup. ATLAS laser energy is split with a diffractive element into six beams, which are grouped into three pairs with pair centers 3.3 km apart (see Fig. 1-1 in Neumann and others, 2022). Each beam pair contains one weak and one strong beam that are 90 m apart. During normal operations over the ice sheets, the central beam pair straddles each reference ground track (RGT; see Table 1 for a complete list of acronyms used in this paper), which is the central of three reference pair tracks (RPTs) associated with each RGT. The RPTs define paths following the centers of the beam pairs when the central beam pair is aligned with the RGTs. Each laser footprint is ~11 m in diameter (Magruder and others, 2021) with a center-to-center separation of 0.7 m in the along-track direction. The paired-beam design resolves both the along-track and across-track components of the surface slope. The use of two different beam strengths allocates the available laser energy to improve the chances that, under cloudy conditions, at least one beam would collect enough photons to allow estimation of the surface height for each pair, while allowing the surface slope to be measured under clear conditions. The relative position of the strong and weak beams reverses approximately every 8 months as the spacecraft is rotated to maintain optimal orientation to the sun, so at times the strong beam in each pair is to the left of the weak beam (when looking in the direction of motion) and at times the reverse. The secondary instrumentation onboard ICESat-2 includes two Global Positioning System receivers, two star tracking cameras and a laser reference system, which provide information for orbit (Thomas and others, 2021) and pointing (Bae and others, 2021) determination. The estimated horizontal geolocation accuracy for the ICESat-2 footprints is generally 3–4 m (Luthcke and others, 2021); this has been confirmed by identifying the returns of the retroreflective targets in the ICESat-2 returns (Magruder and others, 2021) and by comparisons between the elevation of ICESat-2 and the surface topography precisely surveyed (Csatho and others, 2024).

**Table 1.** List of technical acronyms used in this paper. Table 1 long description.

**ATBD**	Algorithm Theoretical Basis Document
**ATLAS**	Advanced Topographic Laser Altimeter System
**DEM**	Digital Elevation Model
**FPB**	First photon bias
**FZ**	Flexure zone
**GZ**	Grounding zone
**HQ**	High-quality
**ICESat-2**	Ice Cloud and land Elevation Satellite—2
**LQ**	Low-quality
**PMT**	Photomultiplier tube
**RGT**	Reference Ground Track
**PT**	Pair Track
**RPT**	Reference Pair Track
**TEP**	Transmit Echo Path
**TPS**	Transmit-pulse shape

ATLAS directly measures heights along 1387 unique RGTs that extend to ±88º latitude. Each ‘cycle’ of ICESat-2 measurements repeats the collection along these RGTs approximately every 91 days (90.8193 solar days, Luthcke and others, 2019) in an exact-repeat orbit. For the Greenland and Antarctic ice sheets, the mission has mostly sampled the same ground tracks on each cycle to provide seasonal resolution of height change. Since ICESat-2 began pointing to the planned RGTs in late March 2019, just after the start of cycle 3 (see Table 2), the deviation of ICESat-2 ground tracks over the ice sheets relative to the reference tracks has been about 20–30 m (Magruder and others, 2025). This variability, on a typical ice-sheet surface with a 1º slope, introduces decimeter-scale apparent height variations from cycle to cycle that can be corrected based on the beam-pair geometry. For sampling specific ‘Targets of Opportunity’, the laser can be off-pointed by up to 10º ∼90 km across-track), and over mid-latitudes it is routinely off-pointed by 1º–2º for vegetation mapping.

**Table 2.** ICESat-2 major events and data gaps. Table 2 long description.

**Date**	**Gap Duration**	**Event**	**Summary**
10/14/2018		Beginning of off-RGT data collection	Initially, the attitude control system in the spacecraft suffered from an error, and measurements are offset from the RGTs by up to several km. Data quality is nominal.
04/01/2019		Beginning of on-RGT data collection	The attitude control system error was fixed, and regular ice-sheet measurements over RGTs began.
06/26/2019	~13.2 days	Safehold	An error in a solar panel orientation sensor caused the spacecraft to orient itself in a ‘safehold’ configuration. Data collection resumed after the error was investigated.
09/10/2021	~1.5 days	Ground Station Complication	A radio link malfunction prevented data download.
01/29/2022	~2.6 days	LRS Reset	A reset of the laser reference system disrupted time tags on instrument-level products, which prevented data from being processed.
04/04/2022	~7.5 days	Safehold	A repeat of the solar position sensor error from 06/26/2019 caused a safehold.
05/04/2024	~1.4 day	Ground Station Complication	A telemetry disruption led to unrecoverable data loss.
05/10/2024	~42 days	Safehold	A solar storm caused additional drag on solar panels, causing the spacecraft to enter the safehold configuration, and the drag on the spacecraft caused a large excursion in the orbit. Data collection resumed after the orbit was restored.

Because ICESat-2 makes dense measurements along its ground tracks, the strongest limitation on the spatial resolution of the mission datasets comes from the across-track distance between adjacent tracks. If ATLAS were a single-beam instrument, this distance would be equal to the distance between adjacent RGTs; however, the left and right PTs (Pair Tracks) in ATLAS’s 6.6 km-wide three-pair beam layout partially fill the spaces between adjacent RGTs. As the RGT spacing decreases toward higher latitudes, the size of the largest gap between measurements varies nonlinearly with latitude (Fig. 1b). Equatorwards of 70º, the largest gaps are between the outer beam pairs on adjacent RGTs (i.e., between pair 3 on one RGT and pair 1 on the next), varying from 3.3 km near 70º to ∼7 km at the equatorward limits of Greenland and Antarctica (60º N and 62º S, respectively). Between 70º and ∼76.5º, the gap between the outrigger pairs on adjacent RGTs is smaller than the 3.3 km gap between ICESat-2’s RPTs, so the largest gap size stays constant at 3.3 km. Poleward of ∼76.5º, the largest gap size fluctuates as the beams interlace, with a prominent maximum at ∼83º where outrigger beams on adjacent RGTs exactly overlap.

ICESat-2 has, to date, operated almost entirely in repeat-track mode over Antarctica, Greenland and the Arctic Basin, with its central beam pair straddling the RGTs. Occasional tracks have been off-pointed to obtain data inside the Antarctic ‘pole hole’, that is, poleward of  88º S latitude. The track sampling transitions to non-repeat-track mode over much of the global land areas outside of the poles to improve the one-time spatial sampling over vegetated areas. For all regions, some data are lost due to sufficiently thick clouds. Maps of the number of repeat measurements over each RPT (Fig. 1d, e) shows nearly 100% coverage (i.e., minimal data lost due to clouds since March 2019) over central Antarctica where thick clouds are rare, with 50–70% coverage (i.e., 30–50% data lost due to clouds since March 2019) over West Antarctica, coastal regions of East Antarctica and much of Greenland. The adjacent part of northern Canada that falls within the repeat mask has rates of data loss due to clouds similar to those of southern Greenland. The rim of the Arctic Basin, where ICESat-2 makes its transitions to and from repeat-track mode, has 30–40% repeat-track coverage, but most tracks farther south have 10–20% coverage, as determined by the vegetation-sampling plan.

### 2.2. ATL03 Level-2 geolocated photon height data

The full-resolution ICESat-2 data is ATL03, Global Geolocated Photons (described in full by Neumann and others, 2019, 2022, 2025). ATL03 provides time-tagged heights for photons telemetered from ATLAS, along with assessments of the confidence that each is a signal photon, geophysical and atmospheric corrections, and instrument parameters. As ATLAS is a green laser (532 nm) in the visible part of the electromagnetic spectrum, high numbers of ‘background’ photons from the sun are present in the data stream during daytime acquisitions. ATL03 provides assessments of the confidence with which photons can be identified as signal photons from two different sets of algorithms: (i) a surface-specific classification method, adapted for land ice, bare earth, and ocean, and (ii) a surface-independent method based on k-nearest neighbors (kNN, Release-06) (Neumann and others, 2022) or radial basis functions (RBF, Release-07) (Herzfeld and others, 2017; Neumann and others, 2025). Geophysical corrections, such as for ocean and solid earth tides, are provided at 20 m spacing along-track. Instrument parameters, such as estimates of the transmit pulse shape as provided by photons collected through the Transmitter Echo Path (TEP), are provided to allow users to derive potential bias corrections for photon aggregates.

In ATL03, every photon and parameter is included, allowing for detailed analysis across all surface types. ATL03 is ideal for examining surfaces and processes that higher-level products do not resolve. Examples of land-ice features that are best investigated with ATL03 are: supraglacial melt ponds, where multiple return surfaces are present (Arndt and Fricker, 2024); crevasses (Herzfeld and others, 2022); and icebergs, which have rough or sharply edged surfaces. However, ATL03 is a large-volume product (Table 3) and typically requires the use of high-performance or cloud computing to access and process data for large (glacier- to ice-sheet scale) regions. Using ATL03 for scientific purposes also requires expert knowledge about the instrumentation and potential measurement biases that have been routinely accounted for in producing the higher-level products.

**Table 3.** ICESat-2 ATLAS data products with their attributes and applications. Table 3 long description.

**Product**	**Name**	**Type**	**Epoch as of 12/2024, mo/yr**	**Data volume^a^**	**Sampling distance and pattern**	**Science application**
**ATL03**	Global Geolocated Photon Data	Individual photons, along-track	10/2018–12/2024	38 TB	0.7 m 1387 RGT × 3 PT × 2 GT	Processes, for example, melt lakes, rifts and crevasses
**ATL06**	Land Ice Height	Along-track average elevations and statistics	10/2018–12/2024	750 GB	20 m 1387 RGT × 3 PT × 2 GT	Processes, dynamic change, grounding zones, subglacial lakes
**ATL11**	Slope-Corrected Land Ice Height Time Series	Along-track height change	10/2018–12/2024	33 GB	60 m 1387 RGT × 3 PT	Processes, large-scale estimates of glacier/ice sheet height change
**ATL14**	Gridded Antarctic and Arctic Land Ice Height	Digital Elevation Model for Antarctic and Arctic land ice	2020.0	1.5 GB	100 m polar-stereo grid	Initialize ice sheet models’ boundary conditions for atmospheric models. Fusion with other satellite data, such as optical imagery or SAR
**ATL15**	Gridded Antarctic and Arctic Land Ice Height Change	Gridded height change estimates	01/2019–12/2024	1 GB	1, 10, 20 and 40 km polar-stereo grid	Visualization of height-change patterns. Calculation of integrated local, regional or continental volume change

a for the area covering the Greenland Ice Sheet, through 12/2024

#### ATL03 limitations

Over highly reflective surfaces, such as ice sheets and standing water, specular reflections can occur, which can partially or completely saturate the detectors and complicate the interpretation of ATLAS returns. ATLAS receives return photons through its telescope and records the return time of each one via photomultiplier tubes (PMTs), of which there are 16 for each strong beam and 4 for each weak beam. Each element and its timing electronics can only detect one photon at any given time, after which there is a time period of about 3 ns during which the element cannot record the return of another photon (typically referred to as ‘dead time’). The photon return rate increases with surface reflectance in the look direction and, for highly reflective surfaces, it is often higher than the system’s maximum recording rate. For high-reflectance returns, the incoming photon rate can be high enough that all the detector elements for a spot are inactive simultaneously, resulting in a 0.5 or 1 m vertical gap in the distribution of recorded photons corresponding to 1 or 2 times the deadtime, followed by a secondary peak corresponding to the reactivation of the detectors. Bright surfaces may also amplify the effect of low-intensity features in the ICESat-2 impulse-response function (Martino and others, 2020), resulting in apparent subsurface reflections at nominal depths of 0.45, 2.36, 4.27 and 6.59 m (see, e.g., Fig. 5 in Arndt and Fricker, 2024). The so-called ‘afterpulses’, from both deadtime and impulse-response, may be misidentified as true sub-surface signals, particularly in applications where two reflections are expected, such as over supraglacial and sea ice melt ponds. Even when afterpulses are not visible in the photon distribution, high photon return rates may bias an aggregation of recorded photons toward those that are first recorded, which is known as the ‘first photon bias’ (FPB; Smith and others, 2019). The FPB is nonzero for most bright ice-sheet surfaces, with typical values on the Antarctic plateau between 0.016 and 0.024 m depending on cloud cover and laser power settings. The FPB and dead time corrections, ffb_corr and rad_corr, respectively, are not applied to photon heights, but are provided in look-up tables on the ATL03 data product and require knowledge of the pulse width and pulse strength.

The ATLAS transmit pulse shape is slightly skewed and deviates from a true Gaussian function (see Fig. 5 in Smith and others, 2019), so that the mean of a sample of photons from near the peak of the distribution may not provide an unbiased estimate of the mean of the entire distribution. This leads to a transmit-pulse-shape (TPS) bias between the mean of any subsample of the return distribution and the mean of the entire distribution, which depends on the surface slope, roughness and the number of background photons mixed with the photons reflected from the surface (Smith and others, 2019). Typical values for the TPS correction on the Antarctic plateau are between 0.008 and 0.018 m.

Modeling studies have demonstrated that subsurface scattering of green light might lead to time-varying biases in ICESat-2 data (e.g., Smith and others, 2018; Henley and others, 2025). Measurements based on airborne laser-altimetry data have shown predicted ICESat-2 biases of several cm over coarse-grained, melting and water-saturated ice surfaces (Fair and others, 2024; Studinger and others, 2024; Smith and others, 2025b), but the distribution of predicted biases derived from airborne measurements spanning the 2019 melt season in Greenland includes very few values larger than 2 cm (Smith and others, 2025b). This suggests that these biases are most likely to be a problem over a limited time range, and over a limited portion of the ice sheets, primarily in the Arctic, where summer melt is more frequent and intense than it is in Antarctica.

### 2.3. ATL06 Level-3A land ice height data

The along-track Level-3A Land Ice Height data product, ATL06, is described in detail by Smith and others (2019). Here, we summarize its characteristics to provide continuity in our discussion of the complete set of land ice products.

ATL06 (version 6 at time of writing; Smith and others, 2023a, 2023e), contains reduced-resolution estimates of surface height derived from ATL03, with height error and measurement quality estimates. ATL06 is a much lower-volume data product than ATL03 (Table 2) and offers the user estimated surface heights with cm-level uncertainties. ATL06 applies an iterative linear fit to photon elevations within 40 m along-track segments to estimate the local surface height and along-track surface slope (see Section 3 and Fig. 3 in Smith and others, 2019). Each iteration progressively isolates the photons within a vertical ‘window’ above and below the sloping surface that spans the assumed linear surface until the selected photons are consistent between iterations. Because the TPS (transmit-pulse shape) bias becomes increasingly large for photons selected from small windows, the algorithm does not allow the window to converge to less than 3 m, bottom to top, limiting the TPS correction to 1–2 cm. After the iterations have converged, the histogram of surface-height residuals around the surface fit is used to estimate both the FPB and TPS bias corrections. For segments in a beam pair that both have valid values, the across-track slope is estimated from the difference between heights. For each ATL06 segment, product variables describe the statistics of photons that contributed to the segment, including the misfit between the photons and the segment, the size of the window, the number of photons in the window, and an estimate of the probability that the observed statistics would result from the algorithm with random-noise inputs. The atl06_quality_summary variable combines these parameters into a binary value that can easily be used to separate high-quality segments from lower-quality segments.

#### ATL06 limitations and alternatives

ATL06 is valuable for users who require details of small-scale features on the order of 50–100 m along track, such as ice fronts (Becker and others, 2021), grounding and flexure zones (Li and others, 2022, 2023; Freer and others, 2023), subglacial lakes (Siegfried and Fricker, 2021; Freer and others, 2024), dolines (Warner and others, 2021) and rifts (Li and others, 2021; Walker and others, 2021; Wang and others, 2021). However, ATL06 may not be straightforward to use for the estimation of height changes over large areas because cross-track displacements in measurement locations from cycle to cycle generate apparent height changes between observations related to surface slope rather than temporal height change. In these cases, the user might be better served using a higher-level product (i.e., ATL11 or ATL15) that corrects for cross-track slope.

The ATL06 algorithm is designed to produce repeatable measurements over smooth ice and snow surfaces. Its 40 m resolution can miss fine-scale features such as crevasses and supraglacial streams, and it does not resolve double surface returns from supraglacial lakes. Options are available to users who need more finely resolved height measurements. Geographical and temporal subsets of ATL03 Level-3A photon-level data can be obtained from the *SlideRule* online processing service (Shean and others, 2023) or from the *OpenAltimetry* subsetting service (Khalsa and others, 2022), and parameters on these products can help segregate background from signal photons. More advanced separation of signal from noise, including identification of multiple surface returns, is possible using more advanced algorithms such as the Density-Dimension algorithm (Herzfeld and others, 2023). Users who need finely resolved surface-height estimates can generate ATL06-like products with customized segment length and along-track posting using *SlideRule*, although users of any of the *SlideRule* products should be aware that they do not include corrections for TPS or FPB biases, so height values may differ from ATL06 by up to 3–4 cm.

## 3. Higher-level land ice data products

**Figure 2.**
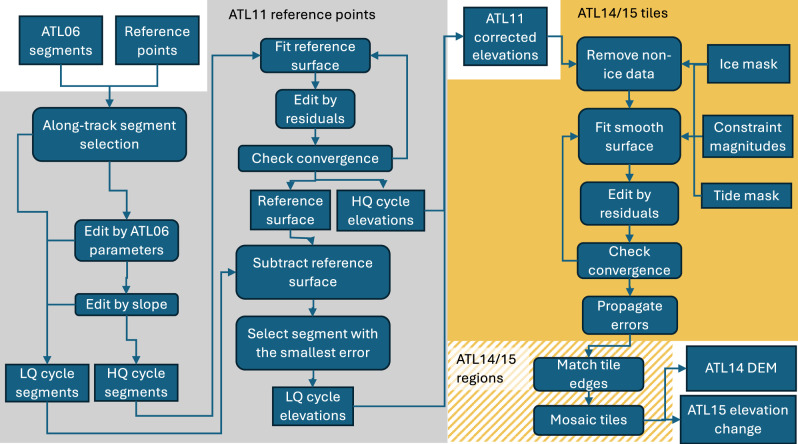
Higher-level product fitting flow chart. Square-cornered boxes indicate intermediate or final data products, and round-cornered boxes indicate processing steps. The gray region indicates the ATL11 fitting process, carried out separately for each ATL11 reference point. The yellow region indicates the ATL14/15 fitting process, where the steps in the solid region are carried out separately for each tile, and the steps in the hatched region are carried out for all the tiles together. Figure 2 long description.

### 3.1. ATL11 Level-3B slope-corrected land ice height data

The Level-3B ICESat-2 Slope-Corrected Land-ice Height Time Series product, ATL11, provides time-varying height estimates for reference points spaced every 60 m along each RPT. Each ATL11 height estimate is derived from ATL06 measurements collected over overlapping 120 m along-track segments, corrected for the small-scale topography within the segment based on a polynomial fit to the ATL06 data. This approach is similar to surface-fitting strategies that have been applied to laser-altimetry data (e.g., Csatho and others, 2014; Schenk and others, 2014), radar-altimetry data (e.g., Nilsson and others, 2022; Khan and others, 2025) or a combination of the two (e.g., Ravinder and others, 2024a), but is adapted to take advantage of the repeat geometry of ICESat-2 measurements. Although similar results might be achieved by correcting ATL06 data based on surface slopes derived from a reference ice-sheet digital elevation model (DEM), ATL11 provides height and height-change data that are independent of the quality or availability of external datasets, using a reference surface derived at a scale consistent with the ICESat-2 measurements.

ATL11 includes height estimates derived from both beams in each pair, distributed across all cycles of the mission, and so it provides the height-change history of a ~120 m × ~90 m area of the ice sheet for each along-track reference point. Where RPTs from different RGTs cross one another, the product also contains measurements from the crossing track, which are similarly corrected for the small-scale surface topography (i.e., ATL11 is both an along-track and a crossover product; Felikson and others, 2017). ATL11 estimates are accompanied by quality flags and error estimates to assist users in assessing data accuracy and reliability. A detailed description of ATL11 processing is provided with the data archive (Smith and others, 2023d) and the algorithm theoretical basis document (ATBD; Smith and others, 2023b). Processing includes three main steps, which are shown as a flow-chart in Fig. 2.

**(i) Data selection and editing:** ATL11 is generated for ICESat-2 tracks that fall within the ATL03 land mask (Neumann and others, 2025), for latitudes of ±60º. For each reference point on each RPT, the ATL11 algorithm collects all ATL06 measurements whose centers fall within a ±60 m search window along the track, and for which the ATL06 algorithm indicates high-quality data (with the atl06_quality_summary parameter). The algorithm then checks the data for self-consistency based on the along-track slope (for each segment) and the across-track slope (for each pair of segments at the same along-track location from the same cycle). We characterize the range of measured slopes around each reference point by calculating the median and *robust spread* (defined as half the difference between the 16th and 84th percentiles of the distribution (Smith and others, 2019)) for all segments within ±60 m of the reference point. Segments that have along- or across-track slopes that are significantly different from the median (i.e., different by more than three times the robust spread for either slope component and more than the segment’s estimated slope error) are marked as invalid. After this check, the algorithm separates the available cycles into two groups: high-quality (HQ) cycles that contain enough consistent measurements to define the across-track slope (i.e., those containing at least two valid segments at the same along-track location), and low-quality (LQ) cycles that do not. Typically, LQ cycles happen when clouds prevent the weak beam in a pair from making usable measurements, so LQ cycles often contain multiple strong-beam measurements.

**(ii) Surface Fitting:** The ATL11 algorithm applies a least-squares fitting procedure to the data from HQ cycles to determine a polynomial reference surface that describes the slope and curvature of the surface around the reference points. This surface is defined in a coordinate system centered on the reference point, with the x-coordinate parallel to the track and the y-coordinate perpendicular. The polynomial degree is chosen based on the number and distribution of available measurements, with a maximum degree of 3 in x and 2 in y. However, due to the small variability in across-track offsets between cycles, the degree in y is almost always limited to 1. This fitting procedure produces a reference-surface polynomial, an error estimate for the reference-surface polynomial, and reference-surface-corrected heights for all HQ cycles.

**(iii) Reference-surface correction:** The ATL11 algorithm uses the reference surface from **(ii)** to correct the data from each valid segment from each LQ cycle and for data from any crossing tracks that intersect the RPT. The algorithm then calculates a formal error for each segment, incorporating the error estimates from the segment and the formal errors in the polynomial coefficients. For each LQ cycle, the algorithm chooses the segment with the smallest error and uses its corrected height to represent the height for that cycle. A similar procedure is applied to obtain reference-surface-corrected heights for data from other RGTs that cross the RGT being processed.

The resulting ATL11 product provides reduced-resolution estimates of changing surface height, with one surface-height estimate for each 91 day cycle, and formal errors for each estimate. By averaging up to 12 ATL06 segments for each estimate and by correcting for the effects of surface shape on cycle-to-cycle differences, ATL11 produces a less noisy estimate of ice-sheet change than can be obtained from direct differencing of ATL06 measurements.

**Figure 3.**
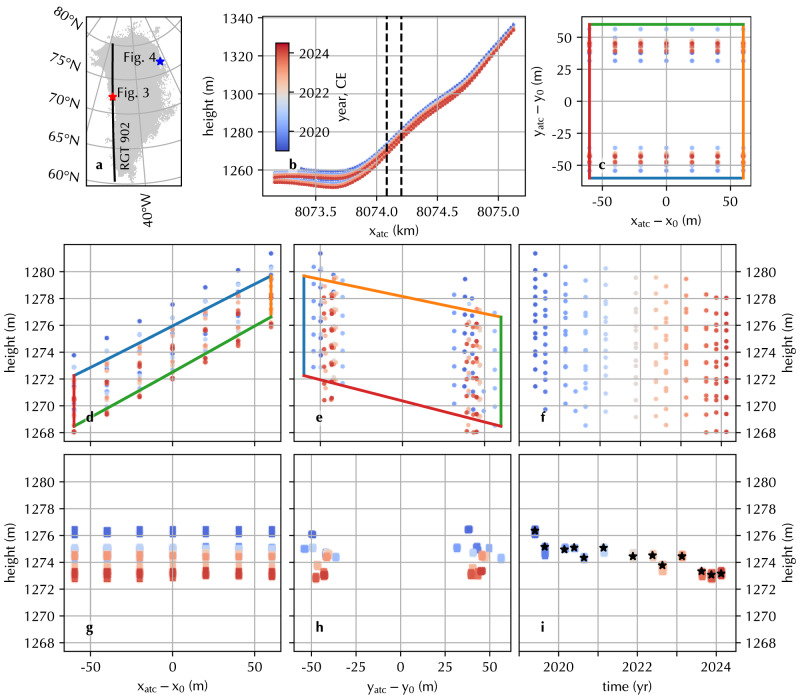
Example of generation of ATL11 from ATL06. (a) track 902 in Greenland. (b) ATL06 measurements for a section of track 902, color-coded by acquisition date. (c) Spatial layout of the measurements as a function of along-track and across-track distance around a reference point with coordinates ($x_{0}$, $y_{0}$). Solid lines show the outline of a 120 m square around the reference point; colors are replicated in (d) and (e) to show the orientation of the reference surface. (d) Uncorrected ATL06 points and the reference surface (line colors matching panel b, shifted vertically to match the mean ATL06 height) plotted against along-track distance. (e) The same uncorrected measurements as panel d, but now plotted against the across-track distance. (f) Uncorrected elevations plotted as a function of time. Finally, ATL06 elevations corrected to the reference surface and shown as a function of (g) relative along-track location, (h) relative across-track location and (i) time, with the ATL11 time series plotted in black on panel i. Figure 3 long description.

We demonstrate the slope correction using the central beam pair from RGT 902 in northeast Greenland (Fig. 3a). We start by showing the height profile for a 2 km section of the track (Fig. 3b), color-coded by acquisition date. We then highlight the layout of measurements around the reference point at x_atc = 8074.16 km in along-track coordinates (Fig. 3c), and we plot a 120 m square around the reference point, with different colors for each side of the square to aid readers in visualizing the shape of the reference surface. There is a ∼3.5º slope in the along-track direction (Fig. 3d) and a ∼2º slope in the across-track direction (Fig. 3e), which leads to apparent scatter in the ATL06 heights when viewed in either direction and also produces substantial scatter when the heights are plotted as a function of time (Fig. 3f). Correcting for the shape of the reference surface reduces the scatter in the heights in both the along-track (Fig. 3g) and across-track (Fig. 3h) direction, leaving most of the substantial remaining variation related to temporal height change (Fig. 3i). The ATL11 corrected height (black stars in Fig. 3i) shows seasonal variations, with large height losses over the summers of 2019 and 2023.

#### ATL11 limitations

The ATL11 dataset was designed to facilitate large-scale assessments of glacier and ice sheet height changes where the surface slope is predominantly static (Fig. 3; Taubenberger and others, 2022; Smith and others, 2023f; Magruder and others, 2024). However, the 120 m resolution may be too coarse for certain applications (e.g., Li and others, 2021; Wang and others, 2021). Additionally, in fast-flowing regions with rough surface topography, such as the crevassed or rifted surfaces typical of dynamic ice sheet margins, we expect repeated height observations of the same location to show substantial height variations due to the advection of small-scale surface features (Moholdt and others, 2014) that may not be well matched by the ATL11 reference surface. In these areas, the user might be better served with careful use of ATL06 (Section 2.3).

### 3.2. ATL14 and 15 Level-3B gridded land ice height data

The ICESat-2 gridded land-ice products provide comprehensive estimates of land ice surface height (ATL14; Smith and others, 2024b) and height changes (ATL15; Smith and others, 2024c) for the Arctic and Antarctic regions, generated from ATL11 (Section 3.1). ATL14 provides a DEM that represents the surface height on 1 January 2020 with 100 m grid spacing that provides a spatially continuous view of the ice surface height and height uncertainty. This DEM can be used for initializing large-scale ice-sheet models and setting boundary conditions for atmospheric models. ATL15 provides quarterly height-change maps relative to the ATL14 reference DEM, evaluated at a coarser resolution of 1 km and also provided at reduced resolutions of 10, 20 and 40 km. These maps are designed to easily visualize height-change patterns, facilitate calculations of integrated regional volume changes, and validate and constrain time-varying numerical ice-sheet models. One advantage of the 10-, 20- and 40-km resolution products is that their error estimates reflect the propagation of correlated (typically per-RPT) errors to these larger spatial scales; the same error propagation cannot be achieved by, for example, smoothing the error estimates on the 1 km products.

The ATL14/15 algorithm (Smith, 2024a) estimates surface height by minimizing a functional that combines the weighted squared misfit between the ATL14/15 model and ATL11 data, the squared magnitudes of estimated data biases and a term penalizing model complexity. This approach balances fidelity to the input observations with smoothness of the resulting height field:

(1) $$R=\sum_{i\in data}{\left(\frac{d_{i}-d_{est,i}}{\sigma_{i}}\right)}^{2}+\sum_{j\in biases}{\left(\frac{b_{j}}{\sigma_{b,j}}\right)}^{2}+F_{0}(z_{0})+F_{\delta }(\delta z)$$

Here $d_{i}$ are the ATL11 heights, $\sigma_{i}$ are the ATL11 error estimates and $d_{est,i}$ are the ATL14/15 estimates of the elevations at each data point plus any biases associated with that data point. For each cycle in the mission, each PT for each RGT is associated with a bias value, $b_{j}$, whose expected value, $\sigma_{b,j}$, is calculated based on the product of the median ATL11 slope for that PT, and the geolocation uncertainty in the measurements. Because the data do not uniquely specify heights for each node in ATL14 and ATL15, the two regularization terms, $F_{\delta }(\delta z)$ and $F_{0}(z_{0})$, are included to penalize rougher surfaces as R is minimized, so that for points that are not well constrained by the data, the ATL14 and ATL15 surfaces are smooth (Parker, 1994). The regularization term for ATL14 ($z_{0}$) is a finite-difference approximation of the integral of the squared second derivatives of the ATL14 DEM plus the squared integral of the DEM gradient:

(2) $$F_{0}(z_{0})\approx \iint amp;\frac{1}{\sigma_{xx}^{2}}[(\partial_{xx}z_{0}{)}^{2}+2(\partial_{xy}z_{0}{)}^{2}+(\partial_{yy}z_{0}{)}^{2}\mathrm{\backslash nonumber}\;amp;+\frac{1}{L^{2}}\left[(\partial_{x}z_{0}{)}^{2}+(\partial_{y}z_{0}{)}^{2}\right]]dA$$

In Eqn (2), $\sigma_{xx}$ is an adjustable parameter that controls the importance of the ATL14 roughness relative to the data-model misfit as R is minimized: Large values of $\sigma_{xx}$ lead to a rougher solution, while small values of $\sigma_{xx}$ lead to a smoother solution. L is a parameter that controls the relative weighting of the first and second derivatives of the solution.

The regularization term for ATL15 is a finite-difference approximation of the integral of the squared second derivatives of the rate of height change plus the squared integral of the gradient of the rate of height change and the second time derivative of the height change:

(3) $$F_{\delta }(\delta_{z})\approx \iint amp;\frac{1}{\sigma_{xxt}^{2}}[(\partial_{xxt}\delta z{)}^{2}+2(\partial_{xyt}\delta z{)}^{2}+(\partial_{yyt}\delta z{)}^{2}\mathrm{\backslash nonumber}\;amp;+\frac{1}{L^{2}}\left[(\partial_{xt}\delta z{)}^{2}+(\partial_{yt}\delta z{)}^{2}\right]]+\frac{(\partial_{tt}\delta z{)}^{2}}{\sigma_{tt}^{2}}dA$$

In Eqn (3), $\sigma_{xxt}$ and $\sigma_{tt}$ are adjustable parameters that control the importance of the roughness of the height-change field and the variability in the rate of change. Two of the adjustable parameters, $\sigma_{xx}$ and $\sigma_{xxt}$, were determined based on an analysis of the horizontal scales of features that should appear in the ATL14 and ATL15 solutions. L was chosen so that gradients in the ATL14 and ATL15 are not extrapolated over long distances (i.e., so that unless trends in $z_{0}$ or $\partial_{t}\delta z$ are present in the data, these fields will be flat), and $\sigma_{tt}$ was chosen primarily to improve the numerical stability of the solution without significantly limiting the variability in δz. The details of these fields are described in the ATL14/15 ATBD (Smith, 2024a). Note that we minimize the derivatives of $z_{0}$ and δz rather than the fields themselves because we do not want to artificially bias the solution toward, for example, small rates of estimated height change; minimizing the derivatives allows us to find smooth solutions that still capture the large-scale features of the ice sheets and their temporal variations. Similar to the ATL11 algorithm, the ATL14/15 algorithm uses an iterative fitting strategy to remove data points that are not statistically consistent with a smooth height variation in space and time. The iteration process is summarized in Fig. 2 and described in detail in the ATL14/15 ATBD (Smith, 2024a).

ICESat-2 only began collecting data over its RGTs at the start of Cycle 3 (29 March 2019); therefore, data from Cycles 1 and 2 are available to the ATL14/15 algorithm only in places where the ground tracks from those cycles cross the RGTs. This results in a sparse sampling of height differences and weak constraints on the spatial pattern of height differences, particularly for Cycle 1 (ending 28 December 2018). Consequently, while elevation measurements from Cycle 1 are ingested to help constrain the surface fit, height differences from Cycle 1 are not included in the final product, and the ATL15 time series begins with Cycle 2. Error estimates for the first surface in ATL15 reflect the increased uncertainty associated with the sparse data coverage in Cycle 2.

**Figure 4.**
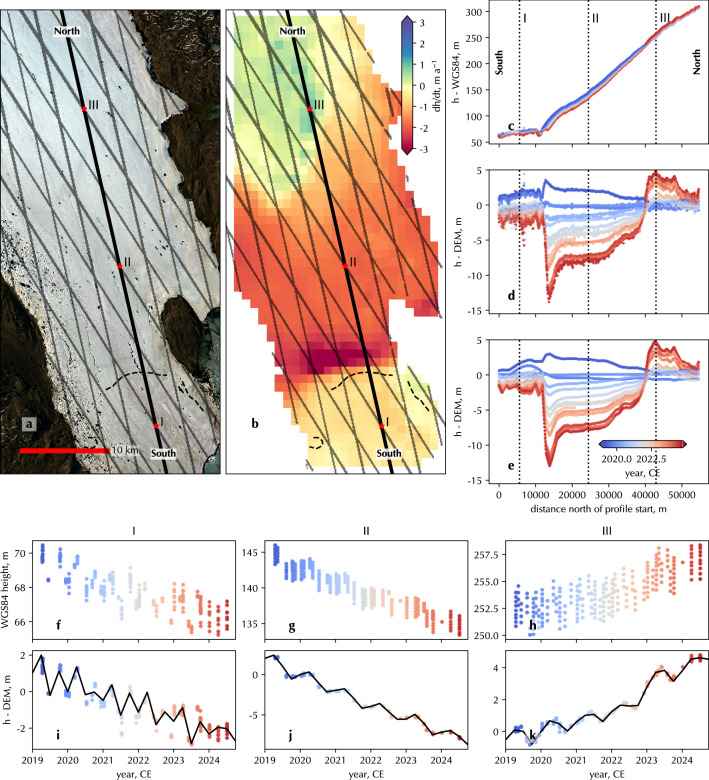
ATL11 to ATL14 and ATL15 processes illustrated at Storstrømmen, Northeast Greenland. (a) Landsat-9 image (LC90542382024199LGN00, 17 July 2024) for a portion of Storstrømmen, Northeast Greenland (see Figure 3a for location), with ATL11 track locations (thin lines) and the 2017 grounding line location (Mouginot and others, 2018, dashed line). (b) Mean rate of height change from ATL15 between 1 January 2019 and 1 April 2024. (c) ATL11 heights from Cycles 3 to 21, color-coded by acquisition year for the profile shown in (a), running south to north. (d) ATL11 heights plotted relative to the 2020 DEM and (e) quarterly ATL15 height differences relative to the 2020 DEM. ATL11 heights collected within 200 m of points (f) I, (g) II and (h) III (see (b) for location) and the corresponding ATL15 time series (black lines) for points (i) I, (j) II and (k) III with ATL11 heights (colored circles) corrected for ATL14 topography. Figure 4 long description.

We demonstrate ATL11-to-ATL15 processing using Storstrømmen Glacier, northeast Greenland (Fig. 4), a surging glacier currently in its quiescent phase (Andersen and others, 2025) that flows into a small floating ice shelf and so provides a spatially varying height change signal. At this latitude (∼79º N), ICESat-2’s RGTs are ~6.6 km apart (Fig. 1b), so the left and right beam pairs for adjacent orbits approximately overlap, and ICESat-2 samples the surface approximately every 30–60 days. Height measurements from ATL11 tracks show that the ice-shelf surface is flat but rough, and that the grounded glacier surface rises gradually to the north (Fig. 4c). For the southernmost 40 km of this profile, surface heights from the early part of the mission are lower than heights from the later part of the mission, implying that the surface is rising over time; northward of 40 km, this pattern is reversed. To demonstrate the spatial variability in the ATL11 data, we removed the large-scale topography from ATL11 by subtracting the ATL14 DEM, leaving height differences that show notable point-to-point scatter (Fig. 4d) that reflect both errors in the ATL11 data and spatial variability in the height change. The recovered quarterly height-differences (Fig. 4e) provide a smoother representation of height change than the ATL11 transects. A map of the mean ATL15 height-change rate (Fig. 4b) shows small surface-lowering rates on the floating ice shelf, much larger lowering rates over a 15–20 km region of the lower trunk of the glacier, and a lobe of thickening ice farther upstream. This likely reflects an actively flowing portion of the glacier moving ice into the stagnant lower trunk of the glacier (Mouginot and others, 2018).

To emphasize the differences between the ATL11 and ATL15 data products, we selected three points along the ATL11 transect (I, II and III, on Fig. 4a). Sampling the ATL11 height time series within 250 m of the three points shows slow surface lowering at I on the floating ice shelf (Fig. 4f), faster lowering at II on the lower part of the grounded ice (Fig. 4g) and slow thickening at point III on the upper grounded ice (Fig. 4h). We corrected ATL11 heights with the ATL14 DEM at I (Fig. 4i), II (Fig. 4j) and III (Fig. 4k), which reduces apparent scatter in the ATL11 heights around each point. For point I on the ice shelf, the ATL15 time series (black line on Fig. 4i) shows substantial scatter, likely because of a combination of errors in the tide correction and topography advecting along the ice shelf. Time series for points II (Fig. 4j) and III (Fig. 4k) each show smaller temporal scatter in ATL15 compared to the raw ATL11 time series (Fig. 4g, h, respectively), which allows separate resolution of the long-term secular height-change rate and the seasonal cycle of height change.

#### 3.2.1. ATL14/15 processing over ice shelves

The ICESat-2 land-ice masks Fig. 1d-e include ice shelves, the floating extensions of glaciers and ice streams that can form where ice sheets meet the ocean. In Antarctica, ice shelves occupy a total area of ~1.5 × $10^{6}$ km${}^{2}$, roughly 10% of the area of the grounded ice sheet. These ice shelves play an important role in ice sheet loss through buttressing (Thomas, 1979; Gudmundsson and others, 2019). In Greenland, only a few small ice shelves (or ‘glacier tongues’) remain (Millan and others, 2023). Over ice shelves, we apply a set of specialized corrections to ATL11 heights that account for the effects of tides, atmospheric pressure variations and ice flexure. To avoid confusion between height changes associated with ice-front advance and retreat and those associated with ice thickness change, we apply a time-varying mask to input ATL11 data and report ATL14 and ATL15 heights only for grid cells identified as ice shelf rather than water. The details of the ice-shelf processing are described in Appendix A.

#### 3.2.2. ATL14/15 limitations

The ATL14 data product is posted at 100 m resolution, but it only resolves features at this scale along RPTs. In the gaps between RPTs, which can be as large as 8 km (Fig. 1b) the resolution of surface features is coarser. The product includes a data_count field that indicates whether each pixel is constrained directly by ATL11 data; those pixels with a data_count value of zero are determined by interpolation of ATL11 observations and should be used with caution. In Appendix B, we evaluate the accuracy of ATL14 by comparing it against scanning laser altimetry data collected by the Operation IceBridge project in 2019 (MacGregor and others, 2021). The product’s accuracy varies with the ruggedness of the surface it is measuring, and with the distance to the nearest ICESat-2 measurement. The 3 month temporal resolution of ATL15 may be insufficient for certain applications, such as measurement of snowfall in ‘atmospheric-river’ events (e.g., Adusumilli and others, 2021). For these applications, the user may wish to create their own grids from ATL06 or ATL11 data, sacrificing spatial sampling (e.g., Ravinder and others, 2024b) or data independence between time slices (e.g., Siegfried and Fricker, 2021) for increased temporal resolution.

For both dynamic grounded ice and ice shelves, lateral advection of features with large vertical amplitudes (e.g., surface crevasses, through-cutting rifts, and roughness features downstream of ice rises and rumples) provides an additional source of temporal height change that can obscure changes in ice volume due to basal and surface mass balance, and strain thinning/thickening. Advection is not accounted for in any ICESat-2 ATL*xx* data products. The effects of advection could be partially mitigated by processing altimetry data in ice-following (‘Lagrangian’) coordinates (e.g., Moholdt and others, 2014), but Lagrangian methods tend to be limited to analyses of track crossovers, losing the high along-track density and repeat-track capability of data from the ICESat-2 mission. Potential future work might include using multi-satellite datasets to incorporate Lagrangian processing into gridded products similar to ATL14/15; however, at the time of writing, gridded surface height changes in these products are calculated in an Eulerian (geographically static) reference frame.

**Figure 5.**
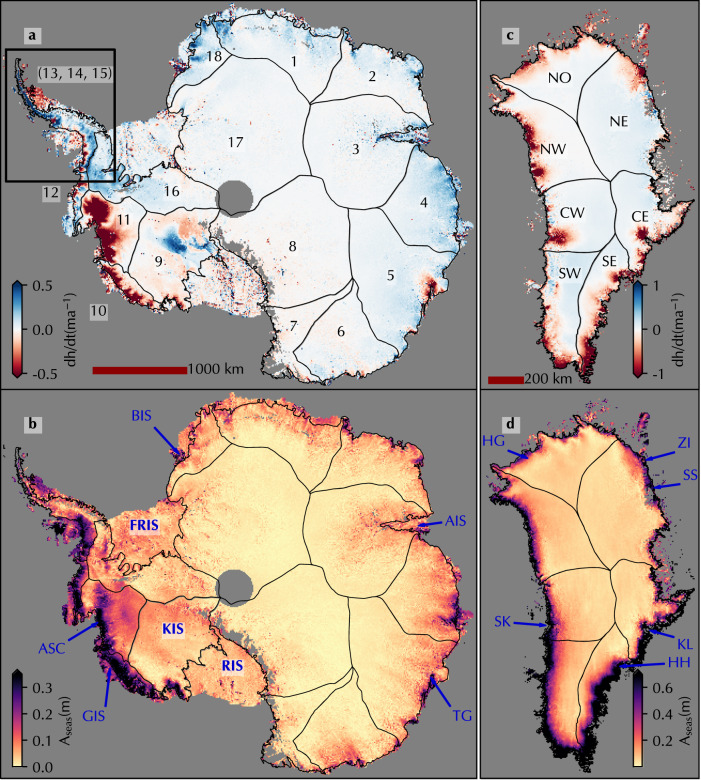
Mean rate of height change and seasonal amplitude for Antarctica and Greenland, January 2019 to December 2024. Panels (a, b) show the rate of height change derived from seasonal models fit to 1 km ATL15 data (Eqn (4) for Antarctica and Greenland, with drainage basin outlines (Mouginot and others, 2017; Mouginot and Rignot, 2019, respectively). Panels (c, d) show the seasonal amplitude derived from the same models. The box in (a) shows the location for Figure 6. Labeled features in (b) are FRIS: Filchner-Ronne Ice Shelf; BIS: Brunt Ice Shelf; AIS: Amery Ice Shelf; RIS: Ross Ice Shelf; GIS: Getz Ice Shelf; ASC: Amundsen-Sea Coast; KIS: Kamb Ice Stream and TG: Totten Glacier. Labeled features in (d) are SK: Sermeq Kujalleq; HG: Humboldt Glacier; ZI: Zachhariae Isstrøm; SS: Storstrømmen glacier; KL: Kanderdlugssuaq Glacier and HH: Helheim Glacier. Figure 5 long description.

Further complications arise in the flexure zone (FZ) of ice shelves near the grounding line (GL), where ice is subject to bending due to tides (Fricker and Padman, 2006; Rignot and others, 2014). For regions where there are low bed slopes and the ice is only lightly or ephemerally grounded, such as on portions of the Ross and Filchner-Ronne ice shelves in Antarctica, the GL, and therefore also the FZ, can migrate several kilometers laterally over the tidal cycle (e.g., Brunt and others, 2010, 2011; Li and others, 2022). Larger tidal range and shallower bed slopes lead to more migration, but migration may also display hysteresis; that is, there may be a different GL location depending on whether the tide is rising or falling (Freer and others, 2023). There are also areas across Antarctica, such as glaciers draining into the Amundsen Sea, that have experienced substantial, multi-km scale retreat of grounding line position in the observational record (e.g., Scheuchl and others, 2016; Verboncoeur and others, 2025). For both of these situations, the applied flexure correction (see Appendix A) will be a poor representation of actual ocean-induced height change.

## 4. Results and discussion

In this section, we demonstrate the spatial and temporal signals of the ICESat-2 higher-order products by analyzing ice sheet height changes during the first 6 years of the ICESat-2 mission (January 2019 to December 2024), focusing on the trend and the seasonal cycle as recorded by ATL15.

Satellite-derived height changes reflect processes that actually affect ice mass (i.e., snow accumulation, meltwater runoff, evaporation and solid ice flux) and processes that do not affect ice mass (e.g., densification via compaction or meltwater retention and refreezing). While it does not affect mass, the densification of snow and firn can lead to substantial height changes that are widespread in space and time and must be accounted for before assessing true ice mass change (Medley and others, 2022; Amory and others, 2024). Several ice-sheet-wide models of the densification process exist (e.g., Ligtenberg and others, 2018; Stevens and others, 2020; Gardner and others, 2023; Thompson-Munson and others, 2023); however, model choice has a non-trivial impact on the results and their interpretation. Therefore, we do not attempt to quantify mass change here. Instead, we keep firn-model interpretation separate and evaluate the height signal directly from the ICESat-2 ATL15 product.

### 4.1. Six-year trends and seasonal amplitudes for Antarctica and Greenland

We summarize some of the most prominent signals in ATL15 by fitting a combined seasonal cycle and linear secular-change model to each 1 km ATL15 pixel in a least-squares sense:

(4) $$dh_{tot}=h_{t}(t-t_{0})+A_{c}\cos\left(2\pi t\right)+A_{s}\sin\left(2\pi t\right)+\varepsilon .$$

Here, $h_{t}$ is the mean rate of height change, $A_{c}$ and $A_{s}$ are amplitudes of the sine and cosine components of the seasonal cycle, t is the time in years, $t_{0}$ is the reference epoch equal to 2020.0 and ε is a residual that is minimized by the least-squares fitting process. For both Antarctica and Greenland, we consider both the long-term signal from the 6 year trends for 2019–25 inclusive (Fig. 5a, b) and seasonal variability, represented by the amplitude of the seasonal cycle; $\sqrt{A_{c}^{2}+A_{s}^{2}}$ (Fig. 5c, d). Note that Fig. 5b, d include locations for geographic features mentioned in the text.

**Figure 6.**
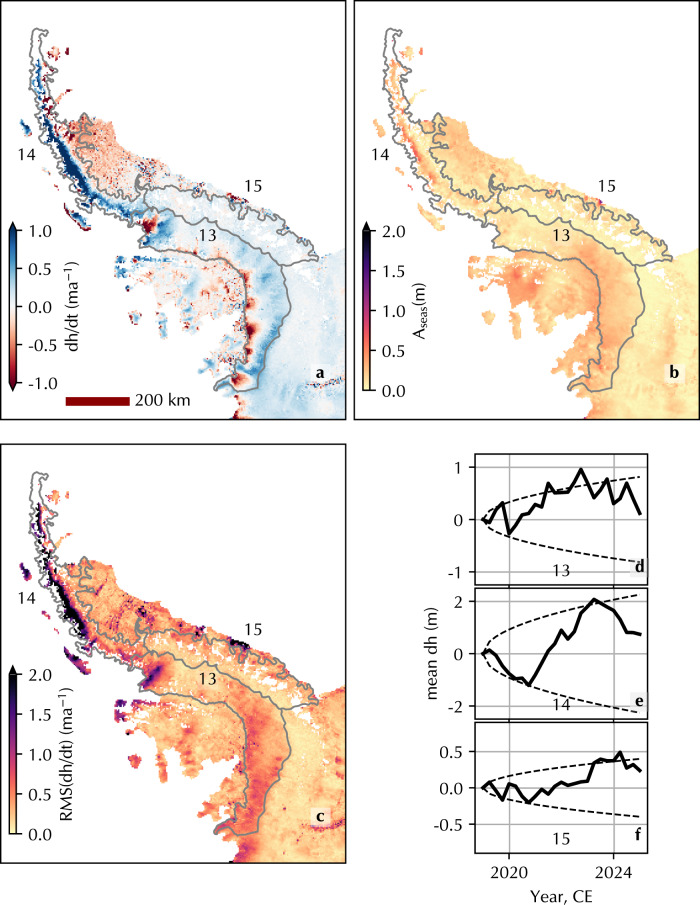
Height change on the Antarctic Peninsula. (a) The mean rate of height change ranges from −2.5 m a${}^{-1}$ near the grounding line of Wordie Glacier at the north end of basin 13 to as much as 2.7 m a${}^{-1}$ on the western slope of region 14 (region outlines from Mouginot and others (2017)). (b) Seasonal amplitudes are commonly greater than 0.5 m and have peak values as large as 1.9 m in region 14. (c) Interannual height-change variability is also large, with values approaching 2.5 m in region 14. Panels (d–f) show mean height changes and expected random-walk changes for basins 13–15. Note that the vertical scale is not the same for (d–f). Figure 6 long description.

For Antarctica, the 6-year trend (Fig. 5a) reveals the effect of sustained surface lowering along the ice sheet margins that flow into the Bellingshausen and Amundsen seas (basins 10–12), attributed to ocean-driven basal melting of ice shelves and consequent acceleration of grounded-ice loss through reduced buttressing (Gardner and others, 2021; Adusumilli and others, 2022; 2023; Fricker and others, 2025). In East Antarctica, thinning occurred in the Totten Glacier catchment (basin 5), consistent with thinning driven by loss of buttressing and grounding line retreat driven by ice-shelf melt (Li and others, 2023). Widespread thickening in East Antarctica is associated with increased precipitation starting in 2022, including contributions from landfalling ‘atmospheric river’ events (Adusumilli and others, 2023; Wille and others, 2024). The largest height increases over the west side of the Antarctic Peninsula locally exceeded 2 m a${}^{-1}$ (detailed in Fig. 6a), consistent with model-based predictions of large mean accumulation rates (Noël and others, 2023) and of SMB-driven height change (Medley and others, 2022; Gardner and others, 2023).

Seasonal amplitude across Antarctica is largest at the coastlines (~0.3 m), decaying to centimeter levels over 100–200 km toward the continental interior (Fig. 5c). Seasonal amplitudes are particularly large along the Amundsen and Bellingshausen coasts (basins 10–12), on the Antarctic Peninsula (basins 13–15), and in parts of coastal East Antarctica (basins 4–6). Seasonal height changes in Antarctica are primarily driven by seasonal variations in snowfall balanced by compaction of fallen snow into firn and ice and by gravity-driven ice flow. Therefore, the patterns of seasonal amplitude likely reflect spatial variations in the rate and seasonality of snow accumulation, and, to a lesser extent, variations in the compaction rate driven by temperature variations. In several areas, seasonal amplitudes show strong orographic control on snowfall; for example, the distinct gradient across north-south-oriented ice ridges in Queen Maud Land (basin 1), where the larger amplitudes on the eastern sides of the ridges likely reflect orographic enhancement of precipitation along the dominant northeasterly storm tracks (Simon and others, 2024). The apparently large localized seasonal signals of height change in the 1 km ATL15 product on the Ross, Filchner-Ronne and Brunt ice shelves are associated with Eulerian height variability owing to the advection of rifts and crevasses.

For Greenland, the main long-term trend is sustained height loss around the margins (Fig. 5b), with strong thinning along the central and southern sectors of the east coast (CE and SE, respectively) and along the Northwest coast (NW). Large rates of surface lowering were also observed for Sermeq Kujalleq (Jakobshavn Isbræ) in the CW sector, for Humboldt Glacier in the NO sector and for Zachariæ Isstrøm in the NE sector. The observed patterns are consistent with a mixture of SMB-driven change and dynamic-driven change associated with accelerating ice discharge through outlet glaciers. The interior of the ice sheet near the flow divide experienced small net thickening, with the largest rates around 0.15 m a^−1^ in SW, consistent with previous observations (e.g., Thomas and others, 2006) and likely associated with a decadal imbalance between precipitation and ice flow in the Greenland interior (Colgan and others, 2015; Kuipers Munneke and others, 2015).

Greenland seasonal amplitudes decay rapidly with distance from the coast in the northern and central parts of the ice sheet, but remain relatively high (0.1 m) at the ice divide in the southern sections (SE and SW; Fig. 5d). The largest seasonal amplitudes approach 0.85 m in the SE, and are much smaller in the NO and NE where the largest amplitudes are around 0.3 m. Unlike in Antarctica, where surface melting makes up a small portion of the surface mass balance, coastal Greenland experiences substantial mass loss due to ice melt and runoff during the summer, and much of the interior experiences summer melt that produces height change by increasing the density of near-surface layers (Medley and others, 2022). Further, many Greenlandic outlet glaciers also exhibit seasonal velocity variations that can drive surface height changes (Moon and others, 2014; King and others, 2018). These processes, together with the larger total accumulation rates in Greenland (Lenaerts and others, 2019), help explain the larger seasonal amplitudes in Greenland compared to Antarctica.

### 4.2. Volume-change rates

We estimated basin-wide and regional volume-change rates (Table 4) from the 6 year trends (2019–25) shown in Fig. 5. For each basin, we estimated the uncertainty in the volume-change rate by propagating the residuals from the seasonal-change fit (Eqn (4)) to determine the uncertainty in the height-change rate for each pixel, then integrating these per-pixel uncertainties to obtain the basin-wide uncertainty. This method provides a conservative (i.e., likely too large) estimate of the basin-wide uncertainty because the per-pixel uncertainties are likely not correlated at the basin scale. We also calculated regional volume-change rates (i.e., for the East Antarctic Ice Sheet (EAIS), West Antarctic Ice Sheet (WAIS), Antarctic Peninsula Ice Sheet (APIS) and Greenland Ice Sheet (GrIS)), and estimated their uncertainty based on the root sum of squares of the basin uncertainties.

**Table 4.** Volume change rate and change rate uncertainties for regions of the Greenland (GrIS) and Antarctic (EAIS, WAIS, APIS) ice sheets. Table 4 long description.

**Ice sheet**	**Region**	**Volumetric change rate** $\left[km^{3}\;a^{-1}\right]$	**Change rate uncertainty** $\left[km^{3}\;a^{-1}\right]$
GrIS	NW	−45	5
	CW	−7	5
	SW	−10	6
	SE	−57	7
	NE	13	5
	NO	−17	3
	Total	−124	13
EAIS	1	39	7
	2	23	6
	3	30	6
	4	64	7
	5	38	15
	6	8	7
	7	−4	3
	8	−2	5
	17	30	9
	18	25	4
	Total	251	24
WAIS	9	−1	8
	10	−44	5
	11	−158	8
	12	−2	2
	16	45	6
	Total	−160	15
APIS	13	9	3
	14	12	3
	15	3	1
	Total	25	4

We emphasize that these volume-change rates are strongly driven by changes in the volume of near-surface firn layers (Medley and others, 2022) and are thus at best roughly proportional to ice-sheet mass-change rates; our volume-change rates are also not corrected for glacial isostatic adjustment or elastic changes in bedrock elevation. Nevertheless, we can use them to examine overall ice sheet change patterns. On the regional scale, GrIS lost volume at a rate of 124 ± 14 km^3^ a^−1^ between 2019 and 2025, with losses concentrated in the NW and SE basins, with small gains in the NE. Over the same time period, EAIS had a substantial volume gain of 251 ± 24 km^3^ a^−1^, with small losses in basins 7 and 8; these basins are notable for having little to no inland height change, in contrast to the other EAIS basins that have inland height gains. WAIS had overall losses at −160 ± 15 km^3^ a^−1^, which are driven primarily by basins 10 and 11, which include the glaciers draining into the Getz Ice Shelf and the Amundsen Sea coast ice streams; this is partially balanced by gains in basin 16, which includes the glaciers draining into the Ronne Ice Shelf. APIS showed small net volume gains in all of its basins.

### 4.3. Short and long-term variability

**Figure 7.**
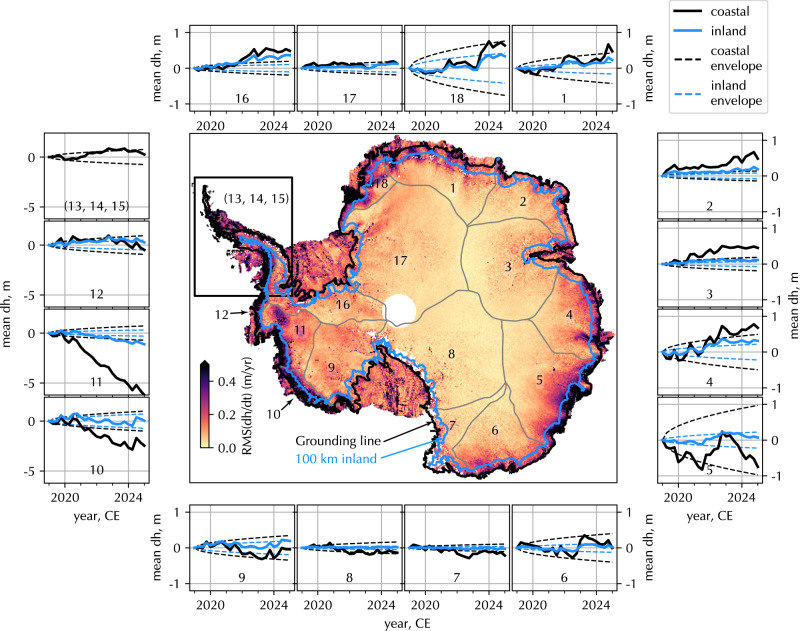
Antarctica seasonal change. Seasonal height-change-rate variability, with time series of height changes for drainage basins around Antarctica (Mouginot and others, 2017). The central panel shows the standard deviation of the height-change rate, calculated on a quarterly basis. Panels around the edge of the figure show the mean height change for individual drainage basins, where we have divided each basin into a coastal band (<100 km from the grounding line measured along flowlines) and the inland remainder of the basin (> 100 km). Figure 7 long description.

**Figure 8.**
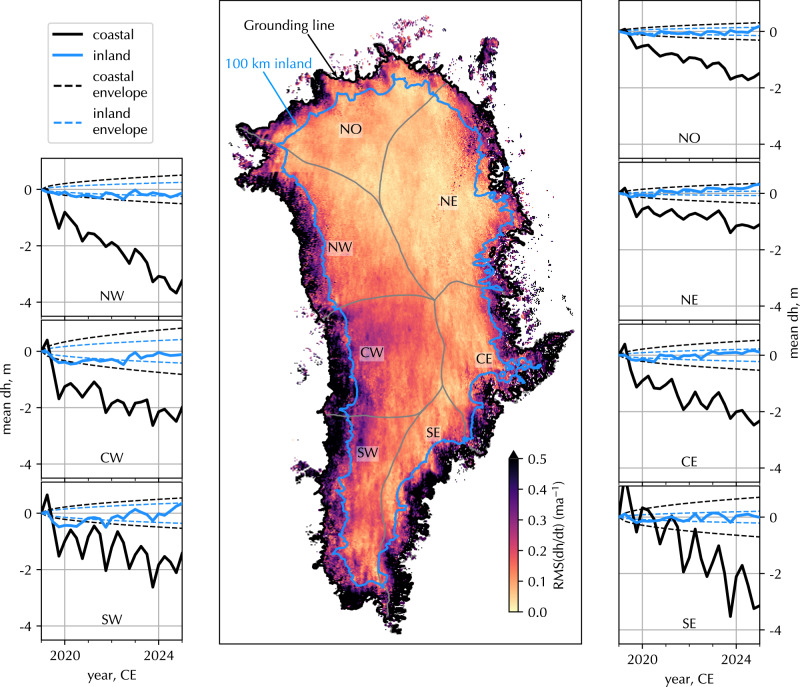
Greenland seasonal change. Seasonal height-change-rate variability, with time series of height changes for drainage basins around Greenland (Mouginot and Rignot, 2019). The central panel shows the standard deviation of the height-change rate, calculated on a quarterly basis. Panels around the edge of the figure show the mean height change for individual drainage basins, where we have divided each basin into a coastal band (<100 km from the grounding line measured along flowlines) and the inland remainder of the basin (> 100 km). Figure 8 long description.

The volume gain and loss observed for Antarctica and Greenland is the net result of complex seasonal variations in surface height. ICESat-2’s 3 month repeat-track sampling resolves these variations at scales of a few km or less. The RMS annual variability in the height-change rate (i.e., the standard deviation among the five annual rates of height change calculated at each pixel) for Antarctica and Greenland (Figs. 7 and 8, respectively; details for the Antarctic Peninsula shown in Fig. 6), gives an estimate of the height-change variability not captured by the mean height-change rate (Fig. 5). For the large-scale drainage basins (Mouginot and others, 2017; Mouginot and Rignot, 2019), the regionally averaged quarterly height changes reveal the temporal patterns of this variability. To account for differences in the sign of the change rate and the magnitude of the spatial variability between the ice sheet edges and interior, we divide each basin into a coastal region, within 100 km upstream of the grounding line along flow, and an inland region, more than 100 km upstream. We exclude the ice shelves since the advection of surface features likely obscures changes driven by dynamics and mass balance.

The regional-average height changes show a mixture of seasonal and interannual changes. Some of these changes are likely due to systematic imbalances between surface accumulation and ice flow that lead to sustained height loss or gain, and others are likely due to short-term fluctuations in surface processes at annual and subannual timescales. To help illustrate the statistical difference between these changes, we calculated the standard deviation in the annual rate of mean height change for each region of each ice sheet (i.e., separating each basin into its coastal and inland regions) and calculated the *random envelope* of change (relative to the start of the mission) that would be expected from an accumulation of random annual changes of this magnitude:

(5) $$dh_{random}=\pm (t-2019{)}^{1/2}\sigma_{1yr}.$$

Here, $\sigma_{1yr}$ is the standard deviation among the five annual rates of height change observed for each region, and (t−2019) is the time since the first epoch in ATL15. Height changes substantially larger than $dh_{random}$ suggest a multi-year imbalance between mean height gain (primarily snow accumulation) and loss (primarily due to melt, compaction and ice flow). This imbalance may be a result of large episodic events such as the 2019 melt event in Greenland (e.g., Tedesco and Fettweis, 2020) or atmospheric river events in Antarctica (e.g., Adusumilli and others, 2021). Alternatively, the imbalance may result from a sustained imbalance between accumulation and ice flow, such as thinning along the Amundsen Coast (e.g., Sutterley and others, 2014; Davison and others, 2023b) or thickening of the upstream Kamb Ice Stream (e.g., Price and others, 2001). Note that in Figs. 6 and 7, we do not divide the basins in the Peninsula into coastal and inland regions because almost the entirety of the Peninsula is less than 100 km upstream of the grounding line.

In Antarctica (Fig. 7), the interannual variability in height change ($\sigma_{1yr}$) rate is typically 1–2 times smaller than the amplitude of the seasonal cycle (Fig. 5). The interannual variability is stronger in coastal regions than in interior regions, although the interior-coastal gradients are not as strong as those seen in the seasonal amplitude. Interannual variability is notably larger in West Antarctica and on the Antarctic Peninsula than in East Antarctica, although coastal parts of basins 4, 5 and 6 show substantial variability.

Comparing regionally averaged net height change (2019–25) to the random envelope calculated from the interannual variability (Eqn (5); sub panels in Figs. 6 and 7) shows that height changes are broadly consistent with the random envelope for both coastal and inland divisions of East Antarctic basins 5, 6, 7, 8 and 17, as well as West Antarctic basins 9 and 12. In basin 5, the 2019–22 height change in the coastal region commonly exceeded the random envelope, but later drifted closer to zero; this pattern shows the value of longer time series of height-change measurements in developing better statistical sampling of interannual signals.

Several basins, particularly in their coastal regions, show net height increases that are clearly in excess of the random envelope. These include East Antarctic basins 1–4 and basin 16 in West Antarctica. The gains are particularly strong in calendar years 2021–23, and may be associated with an enhanced accumulation trend observed starting in 2021 (Adusumilli and others, 2022, 2023). East-Antarctic basin 18 also shows a large gain in excess of the random envelope, although the bulk of this increase came later, in 2023–24. There are also several inland regions where the net height increases exceed the random envelope. These include basins 1–4 and 17–18 in East Antarctica and basins 12 and 16 in West Antarctica.

In contrast to these gains, the entirety of basin 11 (the Amundsen Sea coast, including Pine Island, Thwaites, Smith and Pope glaciers) and, to a lesser extent, the coastal portion of basin 10 (including the glaciers draining into the Getz Ice Shelf) exhibit strong height decreases, well outside their random envelope. Although these basins are known to have large snow accumulation with substantial interannual accumulation variability (Medley and others, 2014; Davison and others, 2023a), the dynamic mass loss, potentially associated with marine ice sheet instability at the grounding lines of these glaciers (Joughin and others, 2014; Rignot and others, 2014), appears to dominate the observed height changes. Basin-averaged height changes for the Antarctic Peninsula basins show large interannual variability, which means that even the 3 m height gain over 2021–23 in basin 14 (Fig. 6) falls within the random envelope.

Greenland (Fig. 8) exhibits moderate (0.2–0.5 m a${}^{-1}$) interannual variability in its coastal region, with smaller inland variability, particularly in the NO and NE regions. The interannual variability in coastal regions is typically a few times smaller than the seasonal amplitude, although the two are often of similar magnitude in the ice-sheet interior.

For Greenland regions, comparing net changes to the random envelope shows small height increases in the inland portions of all basins except CW and NW. These increases are, in most cases, close to the top of the random envelope. In the NW basin, the inland height loss slightly exceeds the random envelope, and in the CW, the loss is very close to zero. The behavior of the coastal regions is sharply different from that of the inland regions. All coastal regions exhibit height decreases far outside the random envelope, with the largest in the NW and SE. Five regions, NW, NE, CW, CE and SW, saw the largest annual height decreases in 2019, corresponding to the 2019 melt event, but the height decreases in other years continue to exceed the lower extent of the random envelope.

## 5. Summary

Since its launch in 2018, NASA’s ICESat-2 laser altimetry mission has been a cornerstone of Earth observation, providing over 6 years of global geodetic surface height data, including over the Antarctic and Greenland ice sheets and land ice in other regions. A range of data products is openly available through the NSIDC, and these data have been used for a wide variety of glaciological studies from processes acting at the smallest resolvable scales (~10 m along-track) to volume change estimates for entire ice sheets. Here, we have described the algorithms that underpin the higher-level data products (ATL11, ATL14 and ATL15), with examples to allow potential users to select the best products for their applications. The seasonal sampling of repeat tracks, albeit with some loss due to clouds (Fig. 1b), provides maps of seasonal surface height change for the ice sheets at high vertical resolution and scales of a few kilometers. When these height data are combined with measured ice velocity and models of firn air content and surface mass balance, they can be used for evaluating mass changes due to ice dynamics (ice flow) and, for floating ice shelves, the basal mass balance due to ocean processes. Continuous acquisition of high-resolution laser altimetry data over land ice is essential to advancing our understanding of the physical processes driving trends and variability in ice sheet dynamics, surface mass balance and glacier change.

## Figures and Tables

**Figure A1.**
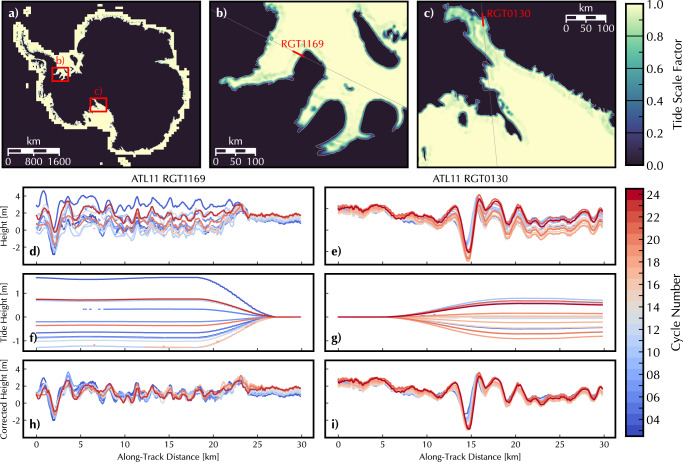
Tide-correction scaling factor to account for ice flexure in (a) all Antarctic grounding zones with detailed views of (b) the southwestern Ronne Ice Shelf and (c) the southern Ross Ice Shelf. Processing chain for ATL14/15 showing ATL11 heights of (d) RGT 1169 (see panel b for location) and (e) RGT 0130 (see panel c for location) with ATL14 topography removed, estimated tidal scaling factor for (f) RGT 1169 and (g) RGT 0130, and ATL11 tide-corrected relative height for (h) RGT 1169 and (i) RGT 0130.

**Figure B1.**
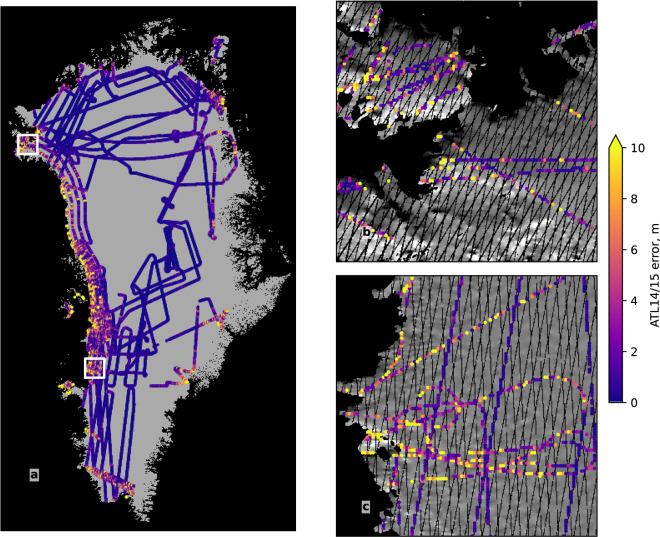
ATL14/15 error estimated for 2 km grid cells around Greenland (a), derived from ATM scanning laser altimetry data collected in 2019, with the ATL15 ice mask for 2019 as a background. ATL14/15 errors (calculated on 1 km grid cells) for two 100 × 100 km example regions (b, c, shown by white boxes in (a)) are plotted over a shaded-relief image of ATL14, with RPTs shown in black.

**Figure B2.**
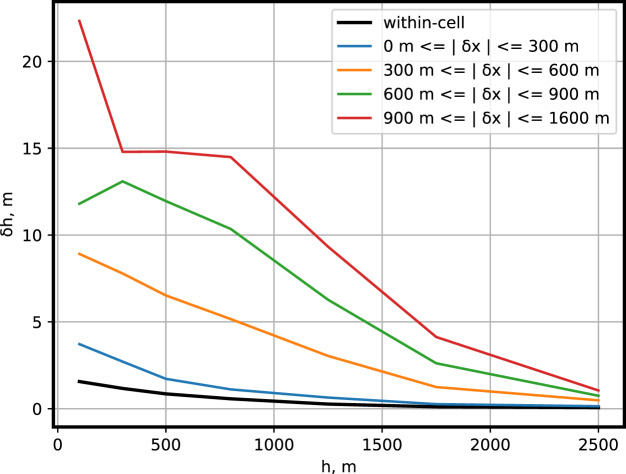
ATL14/15 error estimated for ranges of distances between 200 m cells containing ATM data and the nearest RPT (|δx|), and the median robust spread of the ATM-vs-ATL14/15 differences within the 200 m cells, plotted as a function of surface elevation (h). Differences are calculated only for those 200 m cells containing at least 100 ATM-ATL14/15 difference measurements.

## Data Availability

Software to generate the ICESat-2 higher-level products is openly developed on GitHub, and available from the following links: ATL11: (Smith and others, 2024b git: suzanne64/ATL11), ATL14/15: (Smith and others, 2024a, doi: 10.5281/zenodo.15329774), LSsurf: (Smith, 2024b, doi: 10.5281/zenodo.15330378) and pointCollection: (Smith and others, 2021, git: SmithB/pointCollection). Ice and tide masks used in the generation of the gridded land ice products (ATL14 and 15) are openly available from Zenodo (Smith and Sutterley, 2025).

## Appendix A. Ice-shelf processing

*Ice-front advance and retreat:* The ATL14/15 products are intended to provide surface-height estimates for the ice shelf, and not to include the tens of meters apparent surface-height changes induced as ice shelf fronts advance and retreat (i.e., the difference between the height of the ice-shelf surface in one pass and the sea ice/open water in the next). To mitigate this issue, we apply a time-varying mask. In Greenland, this mask is based on masks developed to separate glacier ice from sea ice for ice-sheet velocity mapping (Joughin, 2023), and is updated quarterly (Black and Joughin, 2022). In Antarctica, it is based on a compilation of ice-front positions with annual resolution until 2022 (Greene and others, 2022), and subsequent quarterly updates based on a combination of machine-learning- derived estimates of ice-front positions (Baumhoer and others, 2023) and hand-digitized front positions based on ESA Sentinel-1 radar imagery obtained from the Alaska SAR Facility (ESA, 2022–2024). As an additional check, at the end of processing, we remove heights that are less than 5 m from the EGM2008 geoid. We use these grids to initialize the ATL14 masks, which cover all 100 m cells that were identified as ice-covered at some time during the mission, and to initialize the time-varying 1 km ATL15 masks, which cover the 1 km grid cells that were identified as ice-covered in each quarter-year epoch. The masks in the coarser resolution ATL15 products are marked as valid for all cells that contain at least one valid 1 km cell, and the ice_area variable describes the area of each cell covered by the 1 km mask, and also accounts for area distortion in the products’ polar-stereographic map projection. The height-change fields in the ATL15 product are marked as valid for time differences that begin and end on a valid cell, and their ice_area fields reflect the area covered in the first and last cell.

*Tide, dynamic atmosphere and flexure corrections:* For portions of the ice shelves that are floating fully hydrostatically, vertical offsets due to sea surface height changes can largely be removed using models for ocean tides (e.g., Padman and Fricker, 2005) and atmospheric forcing (Padman and others, 2003). Modeling is more complicated close to the grounding line (GL), the point at which contact between the ice sheet base and the bed is lost. In the ‘flexure zone’ (FZ; Chen and others, 2023), the ice surface response to ocean forcing is generally damped relative to its hydrostatic value (see, e.g., Fig. 3 in Fricker and Padman, 2006). Ice flexure can be approximated by one-dimensional (1D) elastic beam models that depend on ice strength (Rosier and others, 2017); however, ice strength is poorly understood for damaged (e.g., crevassed) ice and complex GL structure often prevents application of simple 1D flexure models. Where bed slopes are small and/or ocean tide amplitudes are large, ocean variability can also displace the GL up to several km from its mean position over tidal time scales (e.g., Brancato and others, 2020; Freer and others, 2023), with the full range over some short timescale defining a ‘grounding zone’ (Rignot and others, 2024). On longer time scales of months to years, this zone can also migrate and evolve as ice thickness changes. In part because of these complications, there is no readily available model that can predict spatially detailed (km-scale) height variations close to the Antarctic grounding zones.

During ATL14/15 processing, we correct ATL11 heights for the effects of ocean tide fluctuations over ice shelves using an Antarctic regional inverse tide model, CATS2008_v2023 (Howard and others, 2024), and the dynamic atmospheric correction (DAC), a combination of the inverse barometer response (Chelton and Enfield, 1986; Padman and others, 2003) and ocean response to wind stress (Lynch and Gray, 1979; Carrère and Lyard, 2003). In the FZ of Antarctic ice shelves, we derive an empirical correction for the effect of ice flexure on tidal signals based on the correlation between observed variability in height along repeated tracks and the modeled ocean variability for hydrostatic floating ice. For ATL11 tracks crossing the ice shelves and their FZ (example shown in Fig. A1), we calculate the best-fitting scaling between observed vertical height displacements among the repeat measurements at each ATL11 reference point. These scaling factor values are generally close to unity in the interior of the ice shelves far seaward of the FZ, decreasing smoothly to near zero toward the landward side of the GZ. We used a radial basis function (RBF) scheme (Hardy, 1971) to interpolate the reference-point scaling values to a regular 200 m polar stereographic grid, then use this grid to scale the modeled tide and DAC before applying them to the ATL11 height measurements. We generated this correction map based on the data available in early 2022, so the FZ represented does not take into account any potential lateral migration of the GZ (e.g., Milillo and others, 2019; Freer and others, 2024; Rignot and others, 2024; Wallis and others, 2024; Verboncoeur and others, 2025). The correction is applied exclusively in Antarctica; therefore, surface height change estimates in FZs of Arctic ice shelves should be treated with caution as the ATL14/15 algorithm applies no flexure correction.

## Appendix B. Accuracy of the ATL14 DEM

The ATL14 DEM is generated based on a minimum-curvature interpolation of ATL11 surface-height data. Because of ICESat-2’s repeat-track data-collection strategy, the surface is strongly constrained on ICESat-2’s repeat tracks, and much more weakly constrained in the gaps between tracks. However, in many parts of the ice sheet where the surface is flat, a smooth surface interpolated between widely separated measurements can be a good approximation.

To evaluate the accuracy of ATL14 (and to a lesser extent the accuracy of ATL15), we use the scanning laser altimetry measurements carried out in Greenland during the last year of NASA’s Operation IceBridge mission (MacGregor and others, 2021) using the Airborne Topographic Mapper (ATM) instrument (Studinger, 2013). These data provide centimeter-precision measurements of ice-sheet elevation over a 250 m swath below the aircraft, for flights that sampled the ice sheet between April and August of 2019. We reduced the volume of these data and excluded outlying measurements using a 20 m blockmedian separately for each day of the mission (i.e., so that the blockmedian was never applied to data from different days at the same location), and compared these against the sum of the ATL14 DEM and the ATL15 height-change field interpolated to the time and location of the measurements. We then calculated the median and robust spread of the height differences within 200 m grid cells, treating each month of the IceBridge mission separately to avoid mixing true height changes with errors in the data products. We interpret the 200 m-scale differences as follows: The robust spread of differences within any 200 m cell gives an estimate of the small-scale roughness of the ice-sheet surface, which is approximately the sampling error for each ATM measurement. The median difference for each 200 m cell represents one sample of the error in an ATL14/15 approximation of the ice-sheet surface, so the robust spread of median differences, when aggregated over a coarser (2 km) grid, should give an estimate of the accuracy of ATL14/15.

Figure B1 shows the locations of the measurements, color-coded by the robust spread of the median differences aggregated in 2 km cells, and distributions of differences for two sample locations, one in southeast Greenland and the other in northern Greenland, near Humboldt Glacier. The results show decimeter-scale spreads in the ice-sheet interior, with meter-scale spreads in coastal areas, especially in southern Greenland. The insets show that the differences are typically small along the RPTs, increasing in the gaps between RPTs, particularly over rugged terrain in coastal areas. Figure B2 summarizes the dependence between ATL14/15 errors, surface elevation and distance to the nearest RPT (|δx|). In coastal regions (surface elevation < 500 m), ATL14/15 errors range from 2–4 m for ATM cells with |δx| < 300 m to as much as 22 m for points with 900 m < |δx| < 1600 m. In the smoother ice-sheet interior, ATL14/15 errors range from a few cm for points with |δx| < 300 m to 2–3 m for points with 900 m < |δx| < 1600 m.

## Figure 1 Long description

A) Map of Greenland displaying ice velocity in meters per annum, with a gradient from 1 to 1000. Regions i to iv are marked, showing varying speeds. B) Graph of track spacing in meters versus latitude, highlighting RGT-to-RGT and RGT-to-PT gaps. C) Four 10 km by 10 km maps of Greenland locations i to iv, illustrating track patterns with solid and dashed lines. D) Arctic map showing the fraction of valid surface returns from 0 to 100 percent. E) Antarctic map with similar data, indicating surface return validity. The maps and graphs collectively depict ice movement and measurement accuracy across polar regions.

Navigate back to Figure 1.

## Figure 2 Long description

The flowchart illustrates the processing steps for ATL06 segments, ATL11 reference points and ATL14/15 tiles. Starting with ATL06 segments, the process involves along-track segment selection, editing by ATL06 parameters and editing by slope, leading to low-quality and high-quality cycle segments. For ATL11 reference points, the steps include fitting a reference surface, editing by residuals, checking convergence, subtracting the reference surface and selecting the segment with the smallest error, resulting in low-quality cycle elevations and high-quality cycle elevations. The ATL11 corrected elevations feed into the ATL14/15 tiles process, which involves removing non-ice data, fitting a smooth surface, editing by residuals, checking convergence and propagating errors. Additional steps include applying an ice mask, constraint magnitudes and a tide mask. The process concludes with matching tile edges, mosaicking tiles and producing ATL14 DEM and ATL15 elevation change outputs.

Navigate back to Figure 2.

## Figure 3 Long description

Panel A shows a map of Greenland with track 902 marked. Panel B displays height (m) versus xatc (km) from 8073.5 to 8075.0, showing an increase from approximately 1260 to 1340 meters over time, with a color scale indicating years 2020 to 2024. Panel C presents a layout plot of xatc minus x0 (m) versus yatc minus y0 (m), showing two bands around positive and negative 50 meters. Panel D shows height (m) versus xatc minus x0 (m), with heights ranging from 1268 to 1280 meters, indicating a positive slope. Panel E displays height (m) versus yatc minus y0 (m), showing a tilted quadrilateral pattern. Panel F shows height (m) versus an unlabeled axis, with scattered points indicating variability. Panel G presents height (m) versus xatc minus x0 (m), showing consistent height bands. Panel H shows height (m) versus yatc minus y0 (m), with two distinct clusters. Panel I displays height (m) versus time (yr) from 2020 to 2024, showing a slight decline from 1276 to 1273 meters. The panels collectively illustrate different views of the same measurement set, highlighting spatial and temporal variations in height data.

Navigate back to Figure 3.

## Figure 4 Long description

Map with a north arrow near the top and the word South near the bottom. Several thin diagonal lines cross the map. One thicker diagonal line runs from near the top toward the bottom. Three red points on the thick line are labeled I, II and III. A scale bar reads 10 km. Gridded map with several thin diagonal lines and one thicker diagonal line. Three red points on the thick line are labeled I, II and III. The word North appears near the top and the word South near the bottom. A vertical color scale is labeled dh dt in m a superscript minus 1, with tick labels 3, 2, 1, 0, minus 1, minus 2, minus 3. Line plot divided into three sections by vertical dotted lines, with section labels I, II and III. The left side is labeled South and the right side is labeled North. The y-axis label is h minus WGS84, m, with tick labels 50, 100, 150, 200, 250, 300. The x-axis label is distance north of profile start, m, with tick labels 0, 10000, 20000, 30000, 40000, 50000. Multiple colored lines rise from near 50 to near 300 across the distance axis. Line plot divided into three sections by vertical dotted lines, with section labels I, II and III. The y-axis label is h minus DEM, m, with tick labels minus 15, minus 10, minus 5, 0, 5. The x-axis label is distance north of profile start, m, with tick labels 0, 10000, 20000, 30000, 40000, 50000. Multiple colored lines include values near 0 to 5 in section I, values reaching near minus 15 in section II and values rising toward 0 to 5 in section III. Line plot divided into three sections by vertical dotted lines, with section labels I, II and III. The y-axis label is h minus DEM, m, with tick labels minus 15, minus 10, minus 5, 0, 5. The x-axis label is distance north of profile start, m, with tick labels 0, 10000, 20000, 30000, 40000, 50000. Multiple colored lines are shown. A horizontal color bar is labeled 2020.0, 2022.5 and year, CE. Scatter plot labeled I. The y-axis label is WGS84 height, m, with tick labels 66, 68, 70. The x-axis label is year, CE, with tick labels 2019, 2020, 2021, 2022, 2023, 2024. Colored points form a downward pattern from near 70 toward near 66. Scatter plot labeled II. The y-axis label is WGS84 height, m, with tick labels 135, 140, 145. The x-axis label is year, CE, with tick labels 2019, 2020, 2021, 2022, 2023, 2024. Colored points form a downward pattern from near 145 toward near 135. Scatter plot labeled III. The y-axis label is WGS84 height, m, with tick labels 250.0, 252.5, 255.0, 257.5. The x-axis label is year, CE, with tick labels 2019, 2020, 2021, 2022, 2023, 2024. Colored points form an upward pattern from near 250.0 toward near 257.5. Time series plot labeled I. The y-axis label is h minus DEM, m, with tick labels minus 2, 0, 2. The x-axis label is year, CE, with tick labels 2019, 2020, 2021, 2022, 2023, 2024. A black line varies around 0 early and trends downward to near minus 2 by 2024. Colored points are plotted along the time axis. Time series plot labeled II. The y-axis label is h minus DEM, m, with tick labels minus 5, 0. The x-axis label is year, CE, with tick labels 2019, 2020, 2021, 2022, 2023, 2024. A black line trends downward from near 0 to near minus 5 by 2024. Colored points are plotted along the time axis. Time series plot labeled III. The y-axis label is h minus DEM, m, with tick labels 0, 2, 4. The x-axis label is year, CE, with tick labels 2019, 2020, 2021, 2022, 2023, 2024. A black line trends upward from near 0 to near 4 by 2024. Colored points are plotted along the time axis.

Navigate back to Figure 4.

## Figure 5 Long description

A) Map of Antarctica showing the rate of height change in decimeters per year, with values ranging from -0.5 to 0.5. The map is divided into numbered drainage basins. Notable features include the Filchner-Ronne Ice Shelf (FRIS), Brunt Ice Shelf (BIS), Amery Ice Shelf (AIS), Ross Ice Shelf (RIS), Getz Ice Shelf (GIS), Amundsen-Sea Coast (ASC), Kamb Ice Stream (KIS) and Totten Glacier (TG). B) Map of Antarctica showing seasonal amplitude in meters, with values from 0.0 to 0.3. The same drainage basins and features are labeled. C) Map of Greenland showing the rate of height change in decimeters per year, with values from -1 to 1. Regions are labeled as NO, NE, NW, CW, CE, SE and SW. D) Map of Greenland showing seasonal amplitude in meters, with values from 0.0 to 0.6. Labeled features include Sermeq Kujalleq (SK), Humboldt Glacier (HG), Zachhariae Isstrøm (ZI), Storstrømmen glacier (SS), Kanderdlugssuaq Glacier (KL) and Helheim Glacier (HH).

Navigate back to Figure 5.

## Figure 6 Long description

Three maps fill the left of the figure, each showing the same long, narrow, curving peninsula from the same angle but shaded by a different measure. Panel (a) uses a blue to red diverging scale from minus 1.0 to 1.0 for the rate of height change, with a red 200 km scale bar. Panel (b) uses a yellow to purple scale from 0 to 2.0 for seasonal amplitude. Panel (c) uses a similar yellow to purple scale from 0 to 2.0 for the RMS of the height change rate. All three carry outlined regions numbered 13, 14 and 15. On the right, three small line plots (d, e and f) share a horizontal time axis from about 2020 to 2024. Each pairs a solid line for mean height change with a dashed curve for an expected random walk, one plot each for basins 13, 14 and 15, and their vertical scales differ.

Navigate back to Figure 6.

## Figure 7 Long description

The central map of Antarctica displays the RMS height-change rate in meters per year, with a gradient indicating variability. Drainage basins are numbered 1 to 18. A blue line marks the grounding line and a dashed line indicates 100 km inland. Surrounding the map are time series graphs for each basin, showing mean height change (mean dh, m) from 2020 to 2024. Each graph has two lines: coastal (black) and inland (blue), with envelopes (dashed lines) representing variability. The x-axis is labeled ′year, CE′ and the y-axis ′mean dh, m′. Basins 1 to 18 are individually graphed, showing variations in height change over time. Coastal and inland trends are distinct, with some basins showing more variability than others. The legend explains the line types: solid for coastal and inland, dashed for envelopes. The map and graphs together illustrate spatial and temporal patterns of height change across Antarctica′s drainage basins.

Navigate back to Figure 7.

## Figure 8 Long description

Map of Greenland displaying seasonal height-change rate variability with a central panel showing the RMS change rate in meters per year. The map is divided into drainage basins labeled NO, NE, NW, CW, CE, SW and SE. A grounding line and a 100 km inland boundary are marked. The legend indicates RMS change rates from 0.0 to 0.5 meters per year. Surrounding graphs show mean height changes from 2020 to 2024 for each basin, with separate lines for coastal (black) and inland (blue) regions. Solid lines represent actual data, while dashed lines indicate envelopes. The x-axis is labeled ′year, CE′ and the y-axis ′mean dh, m′. Coastal and inland trends are shown for each basin, highlighting differences in height change over time.

Navigate back to Figure 8.

## Table 1 Long description

List intended to define technical acronyms used in the paper by pairing each acronym with its full term or explanation. No rows or columns were provided with the table data, so specific acronyms, definitions, counts, or ordering cannot be described. Because the content is missing, comparisons such as which acronyms appear most often or how they are grouped cannot be confirmed. Provide the table entries to generate accurate that reflects the actual acronyms and definitions.

Navigate back to Table 1.

## Table 2 Long description

ICESat-2 mission milestones and periods of missing data are intended to be listed, typically with event names and associated dates or time ranges. No table entries were provided, so specific events, gap durations, frequencies, or comparisons cannot be described. Without the underlying rows and columns, trends such as clustering of outages or changes over time cannot be assessed. Provide the table values to generate that names the key events, identifies the largest gaps, and summarizes any patterns or caveats.

Navigate back to Table 2.

## Table 3 Long description

ICESat-2 ATLAS data products are intended to be listed alongside their key attributes and common applications. The content would typically map each product name to what it measures or provides, such as elevation, surface characteristics, or derived summaries, and indicate how researchers use it. No rows or columns are included in the provided input, so individual product names, attribute fields, and application examples cannot be described or compared. Any notes about geographic coverage and time limits may affect which products or values apply, but the exact scope cannot be verified without the table entries. Provide the table data to generate precise that summarizes the main products, highlights differences among attributes, and notes any coverage caveats.

Navigate back to Table 3.

## Table 4 Long description

Volume change rates and their associated uncertainties are reported for the Greenland Ice Sheet and for East Antarctica, West Antarctica, and the Antarctic Peninsula. Each region has a numeric rate of volume change along with an uncertainty value, allowing readers to judge both magnitude and confidence. The layout supports direct comparison among the four regions to see which areas are changing fastest and which estimates are most uncertain. Interpretation depends on the sign convention used in the table to indicate loss versus gain, which is not provided here. No specific numbers are available in the supplied data, so relative rankings and exact differences cannot be summarized.

Navigate back to Table 4.
